# Distribution and Molecular Characteristics of *Vibrio* Species Isolated from Aquatic Environments in China, 2020

**DOI:** 10.3390/microorganisms10102007

**Published:** 2022-10-11

**Authors:** Yue Xiao, Zhenzhou Huang, Keyi Yu, Maoshu Wang, He Gao, Xuemei Bai, Mengnan Jiang, Duochun Wang

**Affiliations:** 1 National Institute for Communicable Disease Control and Prevention, Chinese Center for Disease Control and Prevention (China CDC), State Key Laboratory of Infectious Disease Prevention and Control, Beijing 102206, China; 2 Center for Human Pathogenic Culture Collection, China CDC, Beijing 102206, China; 3 National Pathogen Resource Center, China CDC, Beijing 102206, China

**Keywords:** distribution, molecular characteristics, *Vibrio* species, aquatic environment, China

## Abstract

To understand the characteristics of *Vibrio* isolates in aquatic environments in China and their public health significance, this study investigated water samples in six cities in China in 2020. A total of 88 sampling locations were included and *Vibrio* isolates were identified in 81 of them. A total of 143 *Vibrio* isolates belonging to 16 species were selected for characterization. The population structure of *Vibrio* species showed great differences among the six cities, indicating regional specificity. The presence of virulence genes was examined for the isolates (*n* = 78) of five pathogenic *Vibrio* species. All isolates except one (*n* = 77) contained at least one virulence gene and isolates belonging to the same species showed very similar virulence gene profiles. Then, 26 isolates from 12 species were examined by multilocus sequence typing and were assigned to 25 STs, of which 24 STs were new. Also, the presence of antibiotic-resistant genes was investigated for all 143 isolates and only three isolates were found to contain genes from aminoglycosides, phenicols, beta-lactams or the tetracycline family. Our results provide valuable insights into the *Vibrio* community in Chinese aquatic environments and can be applied as guidance for the environmental surveillance of the risk of *Vibrio* isolates.

## 1. Introduction

*Vibrio* spp. are Gram-negative, halophilic, usually motile rod bacteria belonging to the *Gammaproteobacteria* [1]. According to the List of Prokaryotic names with Standing in Nomenclature [2] (LPSN, http://www.bacterio.net, accessed on 28 July 2022), there are 137 validly published *Vibrio* species with correct names, although the recent description of new species may constantly change the taxonomy. *Vibrio* spp. are highly abundant in aquatic habitats, including estuaries, marine coastal water and sediments, and aquaculture settings worldwide. They also appear frequently in and/or on marine organisms, such as corals, fish, and shrimp [1]. Due to the wide distribution and diverse reservoirs, the genus of *Vibrio* comprises of opportunistic pathogenic microbes potentially infecting both humans and animals, posing threats to public health.

Among the pathogenic *Vibrio* species, the most well-known is *V*. *cholerae*. While *V*. *cholerae* with serogroup O1/O139 cause cholera at epidemic and pandemic levels, non-O1/non-O139 *V*. *cholerae* usually cause sporadic cases of diarrhea referred to as cholerae-like illness and extraintestinal tract infection [3]. Non-O1/non-O139 *V*. *cholerae* from marine and estuarine environments can cause human infection by direct transmission [4]. *V*. *parahaemolyticus* is also a well-recognized human pathogen and can cause gastroenteritis [5]. While *V*. *cholerae* and *V*. *parahaemolyticus* can cause severe infections in human and marine organisms, other species such as *V*. *mimicus*, *V*. *harveyi* and *V*. *fluvialis* are also reported to cause illness. *V*. *mimicus* was formerly recognized as a biotype of *V*. *cholerae* but was later reclassified as an independent species due to some differences in biochemical characteristics [6]. *V*. *mimicus* can cause gastroenteritis with symptoms similar to those of *V*. *cholerae*, such as stomachache, diarrhea and nausea [7]. *V*. *harveyi* can cause vibriosis, one of the most prevalent bacterial diseases in marine fish and shellfish, leading to economic losses in the aquaculture industry [8]. *V*. *fluvialis* is an emerging foodborne pathogen and can cause acute diarrhea, gastroenteritis and extraintestinal infection such as hemorrhagic cellulites and cerebritis [9]. Other *Vibrio* species, including *V*. *alginolyticus* [10], *V*. *metschnikovii* [11], *V*. *azureus* [12], *V*. *owensii* [13], are also considered to be pathogenic to marine organisms and/or humans. However, some *Vibrio* species are known as non-pathogenic, such as *V*. *natriegens* [14].

The pathogenicity of *Vibrio* strains is attributed to a broad range of virulence factors encoded by corresponding virulence genes. Virulence factors enable a microorganism to colonize a host niche in which the microorganism proliferates and causes tissue damage or systematic inflammation [15]. Virulence factors not only include secreted proteins and cell-surface structures, which directly invade host cells and contribute to the infection process, but also include gene products such as catalase and regulators, which indirectly involve in pathogenesis. Generally, bacterial virulence factors can be divided into several groups based on the mechanism of virulence and function [16]. For *Vibrio*, there are five major virulence factors, including capsular polysaccharides, adhesive factors, cytotoxins, lipopolysaccharides and flagella [17]. Due to the highly plastic genomes possessed by *Vibrio*, the probability of horizontal transfer of virulence genes is high, which contributes to the evolution, antibiotic resistance and pathogenicity of *Vibrio* community [18]. In recent years, the emergence of multidrug-resistant bacterial strains has become an international health crisis, which poses a significant threat to human well-being. 

The diversity of *Vibrio* strains in aquatic environments leads to an increased interest in analyzing their abundance [19]. 16S rDNA sequencing has been used as an important tool in the accurate identification of bacterial isolates and the discovery of novel bacteria [20]. In addition, multilocus sequence typing (MLST), or multilocus sequence analysis (MLSA), is an efficient tool to achieve genetic characterization and study the molecular evolution of bacterial pathogens [21,22]. In MLST and MLSA, several molecular markers, in single or in concatenated sequences, have been used. Previous studies have used MLST or MLSA to study the phylogenetic relationships of *Vibrio* at the species or genus level [23,24,25]. In order to identify and characterize a population of *Vibrionaceae* isolated from the shellfish of Venice Lagoon (Italy) and to understand the natural diversity of *Vibrio* spp. in that territory, a MLSA scheme was developed based on four housekeeping genes (*recA*, *pyrH*, *atpA* and *gyrB*) and was demonstrated to be very simple and useful for discriminating *Vibrio* species (www.pubmlst.org/vibrio, accessed on 28 July 2022) [26]. 

To under the characteristics of aquatic *Vibrio* species in China and their public health significance, in this study, we investigated 143 *Vibrio* strains from aquatic environments in different cities. The population structure and species diversity of these *Vibrio* strains were analyzed. Also, the virulence gene pattern and the antibiotic resistance profile were examined. Our results provided insights into the characteristics of aquatic *Vibrio* strains in China and the surveillance of pathogenic *Vibrio* isolates. 

## 2. Materials and Methods

### 2.1. Collection of Samples

Water was sampled from six cities (Beijing (B), Fuzhou (M), Qingdao (Q), Qinhuangdao (H), Weihai (W) and Yantai (Y)) in China and a total of 88 sampling locations were included. Apart from Fuzhou, which is located in the south of China, the others are northern Chinese cities. Beijing is a representative of northern inland city while Fuzhou, Qingdao, Qinhuangdao, Weihai and Yantai are representatives of coastal cities. Fuzhou is closed to the East Sea while Qingdao, Qinhuangdao, Weihai and Yantai are closed to the Bohai Sea or Yellow Sea. In Beijing, 12 water samples were collected from 4 rivers (Yongding River, Wenyu River, Sha River and Qing River), which cover the northern city waterway. In Fuzhou, 25 water samples were collected from Min River, which is the largest river in Fujian province. In Qingdao, Qinhuangdao, Weihai and Yantai, 51 samples (Qingdao: 12 samples; Qinhuangdao, 9 samples; Weihai: 18 samples; Yantai: 12 samples) were collected from aquatic places in the populous areas of each city. The water samples mentioned above were collected over a period of two months (September 2020–October 2020). September and October were selected because of warm weather and stable temperatures, which contribute to *Vibrio* abundance. 

### 2.2. Isolation and Identification of Presumptive Vibrio Species

Each water sample was enriched in alkaline peptone water and then incubated at 37 °C for 18–24 h. A loopful from the alkaline peptone water was inoculated onto ten thiosulphate citrate bile salts sucrose (TCBS) agar plates and incubated for another 24 h at 37 °C. Four to six suspected colonies of *Vibrio* were randomly chosen from each plate and consequently purified on fresh LB agar plates. Then, the purified colonies were detected by mass spectrometry analysis using MALDI-TOF MS EXS3000 (Zybio Inc., Chongqing, China) to select presumptive *Vibrio* isolates. 

### 2.3. Genomic DNA Extraction and Vibrio Isolates Confirmation

The purified presumptive *Vibrio* colonies were inoculated in LB medium and incubated at 37 °C for 18–24 h. The overnight bacterial suspension was used to extract genomic DNA by Wizard Genomic DNA Extraction Kit (Promega, Madison, WI, USA) following the manufacturer’s instructions. The genomic DNA was quantified by NanoDrop 2000 spectrophotometry (Thermo Fisher Scientific, Waltham, MA, USA) and then used as the template of polymerase chain amplification (PCR) assay, with the universal primers (27F/1492R) of 16S rRNA gene [27]. The PCR products were then sequenced using the same primers. Subsequently, the sequences were searched by the Basic Local Alignment Search Tool (BLAST) [28] of The National Center of Biotechnology Information (NCBI) database for *Vibrio* species identification. To further confirm the *Vibrio* isolates and to classify these isolates into species, a phylogenetic tree was constructed with the 16S rRNA gene sequences of the isolates and valid type strains from The List of Prokaryotic names with Standing in Nomenclature (LPSN) [2] by MEGA 7.0 [29], using the neighboring–joining method. The selected model was Kimura’s two-parameter with partial-deletion (95%) option. The robustness of tree topologies was evaluated with 1000 bootstrap replications. Then, the confirmed *Vibrio* isolates were chosen for further experiments based on the principle that for one species, only one strain from one isolation place was selected. 

### 2.4. Detection of Virulence Genes

Virulence genes in the isolates (*n* = 78) of five common pathogenic species (*V*. *cholerae*, *V*. *mimicus*, *V*. *parahaemolyticus*, *V*. *harveyi* and *V*. *fluvialis*) were identified through a polymerase chain reaction (PCR) assay. The targeted virulence genes in *V*. *cholerae* include *ctxAB*, *tcpA* (1), *tcpA* (2) (two pairs of *tcpA* primers were used to amplify divergent *tcpA* alleles in the 7th pandemic *Vibrio* pathogenicity island (VPI)), *mshA*, *hlyA*, *rtxC*, *rtxA*, IS1004, *chxA*, SXT, T3SS (vcsV2) and *nag*-*st*. The targeted virulence genes in *V*. *mimicus* include *vmh*, *tdh*, *hlx* and *st*. The targeted virulence genes in *V*. *parahaemolyticus* include *tl*, *tdh* and *trh*. The targeted virulence genes in *V*. *harveyi* include *luxR*, *toxR_Vh_*, *chiA*, serine protease and *vhh*. The targeted virulence genes in *V*. *fluvialis* include *vfh*, *hupO* and *vfpA*. Primers, their annealing temperatures, and amplicon sizes are listed in Table 1. The PCR products were assessed with 1.0% agarose gel electrophoresis.

### 2.5. Multiple Locus Sequence Typing (MLST)

MLST was performed to determine the phylogenetic relationships and genetic heterogeneity among these *Vibrio* isolates. PCR assays targeting the four housekeeping genes (*gyrB*, *pyrH*, *recA* and *atpA*) of *Vibrio* genus were performed for 26 isolates, which belong to 12 different species. Primers, their annealing temperatures, and amplicon sizes are listed in Table 1. The PCR fragments were sequenced and analyzed by comparison with sequences obtained from PubMLST database (https://pubmlst.org/organisms/vibrio-spp, accessed on 28 July 2022) [26,50]. PHYLOViZ version 2.0, using the goeBURST algorithm [51], was used to calculate and visualize clonal complexes (CCs) between STs of isolates in this study and STs from PubMLST database. 

### 2.6. Detection of Antibiotic-Resistant Genes

PCR assays were performed in all isolates (*n* = 143) to identify various antibiotic-resistant genes. The selected targeting genes include aminoglycosides resistance genes (*strA*, *strB*, *addA*, *aac(3)-IIa* (*aacC2*), *aph(3’)-Ia* (*aphA1*), *aph(3’)-IIa* (*aphA2*)), chloramphenicol resistance genes (*cmlA1*, *catI*, *catII*, *floR*), beta-lactamase gene (*bla*_TEM_), sulfonamide resistance gene (*sulI*), trimethoprim resistance gene (*dfrA1*), quinolone resistance gene (*qnrA*), tetracycline resistance gene (*tetA*). Primers, their annealing temperatures, and amplicon sizes are listed in Table 1. The PCR products were assessed with 1.0% agarose gel electrophoresis. 

## 3. Results

### 3.1. Population Structure and Regional Distribution of Vibrio Isolates

**Figure 1:**
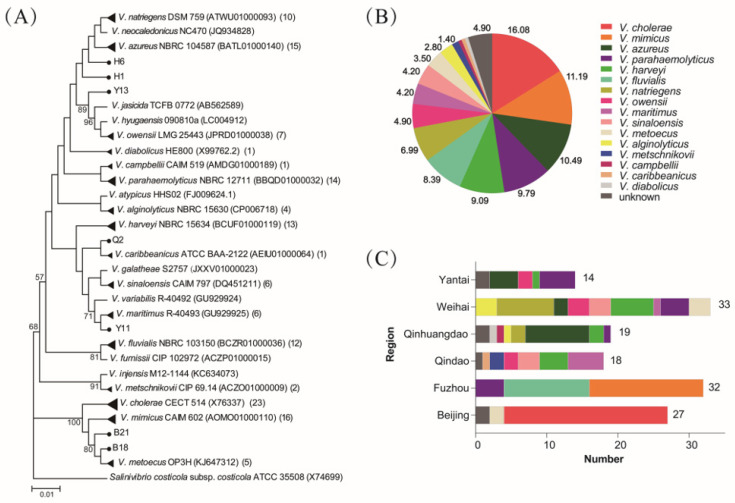
The *Vibrio* species composition and regional distribution: (**A**) the phylogenetic tree reconstructed by neighbor-joining method based on genes of 16S rRNA. *Vibrio* type strains were involved and accession numbers of 16S rRNA sequences were shown in the adjacent parentheses. Strain number of tested strains for each compact cluster (black triangle) was shown in the final parentheses. Unclassified tested strains were indicated by black circles. Bootstrap values were calculated from 1000 replications and values >70% were shown at branch points. Bar and value estimated nucleotide substitutions per site. The type strain of *Salinivibrio costicola* subsp. *costicola* ATCC 33508 was served as an outgroup; and (**B**) species composition (%) of *Vibrio* isolates. (**C**) Regional distribution of *Vibrio* isolates.

Among the 88 sampling locations, *Vibrio* have been identified in 81 (92%) of them (Beijing: 9/12; Fuzhou: 22/25; Qingdao: 12/12; Qinhuangdao: 9/9; Weihai: 17/18; Yantai: 12/12). A total of 2548 bacterial strains were isolated, in which *Vibrio* strains accounted for 36% (917). The rates of *Vibrio* strains in Beijing, Fuzhou, Qingdao, Qinhuangdao, Weihai and Yantai were 22% (283/1261), 33% (130/399), 46% (122/267), 61% (103/169), 61% (158/260) and 63% (121/192), respectively. Based on the principle that only one strain from one sampling location was selected for one *Vibrio* species, a total of 143 *Vibrio* strains, which belong to 16 species according to 16S rRNA sequence, were selected. Specifically, 23 *V. cholerae*, 16 *V. mimicus*, 15 *V. azureus*, 14 *V. parahaemolyticus*, 13 *V. harveyi*, 12 *V. fluvialis*, 10 *V. natriegens*, 7 *V. owensii*, 6 *V. maritimus*, 6 *V. sinaloensis*, 5 *V. metoecus*, 4 *V. alginolyticus*, 2 *V. metschnikovii*, 1 *V. campbellii*, 1 *V. caribbeanicus* and 1 *V. diabolicus* were identified, accounting for 16.08%, 11.19%, 10.49%, 9.79%, 9.09%, 8.39%, 6.99%, 4.90%, 4.20%, 4.20%, 3.50%, 2.80%, 1.40%, 0.70%, 0.70% and 0.70% of the total strains, respectively (Figure 1A,B). Notably, seven strains failed to be identified at the species level by 16S rRNA sequences and were classified as unknown (4.90%). 

The composition of *Vibrio* species showed diversity among different cities (Figure 1C). Some species showed specificity towards regions. Strains of *V. cholerae* were isolated exclusively in Beijing, while those of *V. mimicus* and *V. fluvialis* were isolated exclusively in Fuzhou. Strains of *V. metschnikovii* and *V. caribbeanicus* were identified only in Qingdao, while those of *V. campbellii* and *V. diabolicus* were identified only in Qinhuangdao. For the isolates from Weihai, nine different species were found. By contrast, for the isolates from Beijing, only two different species were identified. 

### 3.2. Distribution of Virulence Genes in Vibrio Isolates

**Figure 2:**
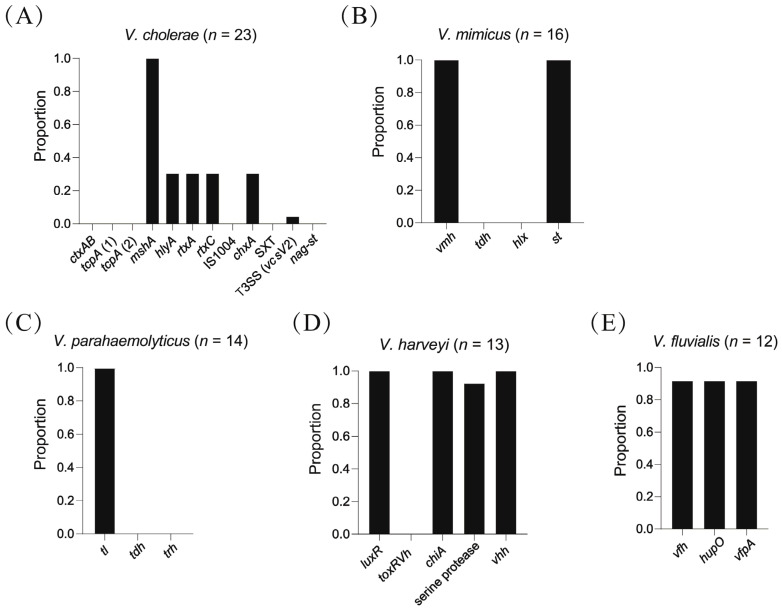
Distribution of virulence genes among five common pathogenic species. The bars represent the proportions of virulence genes in each species (**A**–**E**).

The virulence genes for five pathogenic species (*V. cholerae*, *V. mimicus*, *V. parahaemolyticus*, *V. harveyi* and *V. fluvialis*) were identified in corresponding isolates (*n* = 78). All isolates except one (*n* = 77) contained at least one virulence gene. Isolates belonging to the same species showed very similar virulence gene profiles. For the isolates of *V. cholerae* (Figure 2A), all harbored *mshA* gene, but *ctxAB*, *tcpA* (1), *tcpA* (2), IS1004, SXT and *nag-st* genes were not identified. Nearly one third of the *V. cholerae* isolates (7/23) harbored *hlyA*, *rtxC*, *rtxA* and *chxA* genes, accounting for 30.4% of the total isolates. Only one strain (4.3%) harbored T3SS (*vcsV2*) gene. For the isolates of *V. mimicus* (Figure 2B), all harbored *vmh* and *st* genes, but *tdh* and *hlx* genes were not identified. For the isolates of *V. parahaemolyticus* (Figure 2C), all harbored *tl* gene, but *tdh* and *trh* genes were not identified. For the isolates of *V. harveyi* (Figure 2D), all harbored *luxR*, *chiA* and *vhh* genes, but *toxR_Vh_* gene was not identified. Also, all *V. harveyi* isolates except one (12/13, 92.3%) harbored the gene encoding serine protease. For the isolates of *V. fluvialis* (Figure 2E), all except one (11/12, 91.7%) harbored *vfh*, *hupO* and *vfpA* genes. 

### 3.3. MLST Analysis of Vibrio Isolates

A total of 26 isolates, belonging to 12 species, were typed by MLST and were assigned to 25 STs (ST_24_ and ST_472_–ST_495_), of which 24 STs were new (ST_472_–ST_495_) (Table 2). For the alleles, 22 alleles of *gyrB* gene (223–244) were new, followed by 20 of *recA* gene (216–235), 16 of *pyrH* gene (158–173), and 13 of *atpA* gene (159–171). The ST_24_, which was not new in this study, was also found in live marine animals in Italy based on data from the PubMLST database [26,51] (https://pubmlst.org/organisms/vibrio-spp, last accessed 7 June 2022). The ST_484_, which was identified for the first time in this study, consists of previously identified alleles. Apart from ST_484_, other new STs consist of new alleles.

**Figure 3:**
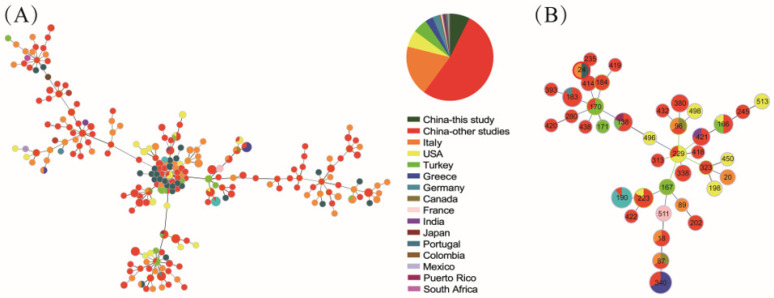
The goeBURST analysis based on the four loci used for MLST typing: (**A**) the full minimum spanning tree generated by 223 STs (including the 25 STs identified in this study) from pubMLST database for *Vibrio* spp. Isolates from different countries are represented by different colors. Node sizes are proportional to the number of isolates; (**B**) clonal complex 0 of 40 STs. STs are marked inside the circle. The ST_24_ identified in this study is marked by red circle.

We further analyzed the 25 STs using goeBURST algorithm [52] within PHYLOViz [51] to identify clonal complexes (CCs). The formation of CCs is based on the definition that all STs in the same clonal complex should share at least three MLST loci with at least one member from the identical clonal complex. A total of 351 isolates from different countries [26,50], including the 26 isolates in this study, were included. A total of 223 STs were found among these isolates. A full minimum spanning tree (MST) was generated (Figure 3A). 152 CCs were identified, in which 132 CCs were singleton. For the 25 STs assigned by 26 isolates in this study, 24 STs (ST_472_–ST_495_) were singletons, while ST_24_ belonged to CC0, which was the biggest CC and contains 40 STs (Figure 3B).

### 3.4. Detection of Antibiotic-Resistant Genes in Vibrio Isolates

Resistant genes encoding aminoglycosides, chloramphenicol, beta-lactamase, sulfonamide, trimethoprim, quinolone, and tetracycline, were examined in this study. Aminoglycosides resistance genes *strA*, *strB*, *aadA*, *aph(3’)-Ia* (*aphA1*), chloramphenicol resistance genes *cmlA1*, *floR* and tetracycline resistance gene *tetA* were identified in B19. The above genes, except *aadA*, *aph(3’)-Ia* (*aphA1*) and *cmlA1*, were also identified in B21. Beta-lactamase gene *bla*_TEM_ was identified in W32. In other isolates, no antibiotic-resistant gene included in this study was identified.

## 4. Discussion

*Vibrio* spp. usually inhabit in estuarine and marine ecosystems and can cause severe infections in humans and animals. Although the presence of pathogenic *Vibrio* isolates in aquatic environments is gaining attention, the population structure of aquatic *Vibrio* isolates in Chinese cities is largely unknown. Here, we studied 143 *Vibrio* isolates collected from different aquatic environments in six cities in China, aiming to understand the distribution and characteristics of these *Vibrio* strains.

Among the six cities, a total of 88 sampling locations were included and *Vibrio* isolates were detected in 81 of them (92%). The isolation rates of *Vibrio* in each city were not compared because the numbers of sampling location in each city were small. In a previous study characterized the population structure of non-O1/non-O139 *Vibrio cholerae* in fresh rivers in Zhejiang, China, the average isolation rate was 43.9% [53], which is different from our study and can be explained by the differences of geographic factors and focused species. Among the 143 *Vibrio* isolates, 16 different species were identified, demonstrating abundant and diverse *Vibrio* populations in Chinses aquatic environments. The diversity of *Vibrio* community in northern Chinese marginal seas has been confirmed [54], which is consistent with the abundance of *Vibrio* isolates in the northern coastal cities included in our study. In these cities, the average isolate rate of *Vibrio* was 99%. Also, a study about *Vibrio* populations from rustic freshwaters recovered substantial isolates from nine sampling sites [55], suggesting inland freshwater environments are also ideal habitats for *Vibrio* species.

*Vibrio* isolates were detected through molecular analysis of 16S rRNA gene and were classified into species through a phylogenetic tree. Previous studies have employed similar methods to confirm *Vibrio* spp. strains [17]. Although several *Vibrio* species have nearly identical 16S rRNA sequences, the nearly complete 16S rRNA sequences are likely to give accurate measures of taxonomic diversity [56]. Our subsequent analysis of virulence gene confirmed the correct species classification of most *Vibrio* isolates. We identified 16 known species among all *Vibrio* isolates, indicating the diversity of *Vibrio* community in aquatic environments in China. *Vibrio* populations in coastal cities were more abundant than those in inland cities, suggesting salinity is a pivotal environmental determinant of the pattern in *Vibrio* community [57]. The abundant and diverse *Vibrio* populations have been demonstrated in northern Chinese marginal seas [54], where *V*. *campbellii* and *V*. *caribbeanicus* were shown to be the most prominent in summer, while *V*. *atlanticus* was the most isolated in winter. A study focused on the spatiotemporal dynamics of *Vibrio* communities and abundance in Dongshan Bay, an important aquaculture base in southern China, have identified 28 species among 167 *Vibrio* strains in 10 seawater sites spanning four seasons [58]. In this study, the *Vibrio* isolates were collected from different aquatic environments in populous cities, which may explain the differences observed in *Vibrio* species distribution. Multiple environmental factors, including temperature, salinity, pH, dissolved oxygen and nutrients contribute to the composition of *Vibrio* population [57]. Among them, temperature and salinity are considered to be the most important while other variables often have a marginal effect. Previous studies have demonstrated *Vibrio* populations in marine aquaculture are more abundant in summer than in winter [54,58], which suggests temperature is a key factor that influences *Vibrio* abundance. However, in summer (July and August), the temperature of surface water in aquatic environments is high so *Vibrio* abundance across sites may exhibit few variations [54]. In this study, aquatic samples were collected in September and October (the end of summer and the beginning of autumn), when the temperature is more stable, and the weather is still warm. Our results indeed showed great variations across different sites. Among the four coastal cities, the dominant *Vibrio* species were different. Further studies are needed to explore the environmental factors which influence the population structures of *Vibrio* in these cities.

The identification of virulence genes in *Vibrio* strains is important to evaluate their pathogenicity. The virulence genes in five common pathogenic *Vibrio* species were examined in this study: (1) for *V*. *cholerae*, the major virulence factors are toxin-coregulated pili (TCP) and cholera toxin (CTX), which are present in all pandemic strains. All isolates (*n* = 23) of *V*. *cholerae* in this study were negative for *ctxAB*, *tcpA(1)* and *tcpA(2)*, indicating they should be non-O1/non-O139 strains. The genes IS1004 encoding an active mobile genetic element [59], SXT encoding an integrative conjugative element carrying multiple drug resistance gene [60] and *nag*-*st* encoding heat-stable toxin [61], were also absent in these isolates. Other virulence genes including genes encoding the cytotoxic actin cross-linking repeats in toxin (*rtxA*, *rtxC*), cholera toxin transcriptional activator (*toxR*), hemagglutinin/protease (*hapA*), hemolysin (*hlyA*), mannose-sensitive hemagglutinin (*mshA*), a type 3 secretion system (T3SS), are also common in *V*. *cholerae* strains [53]. The T3SS has been shown to play a critical role in colonization and causing diarrhea in strains without TCP and CTX [62]. In this study, the *mshA* gene was identified in all isolates, while the *hlyA*, *rtxA*, *rtxC*, *chxA* and T3SS (*vcsV2*) genes were positive for some isolates, with the ratios of 30.4%, 30.4%, 30.4%, 30.4%, 30.4% and 4.3%, respectively. The *V*. *cholerae* isolates (*n* = 23) were only identified in Beijing, which may explain the homogeneity of virulence gene profiles. The proportions of these virulence genes were consistent with a previous study [61] about the non-O1/non-O139 *V*. *cholerae* aquatic isolates from China, in which the *mshA* gene was the most common while the T3SS gene was the least. (2) For *V*. *mimicus*, the major virulence factors are three types of hemolysins, encoded by *vmh*, *tdh*, *hlx* genes, respectively [38]. *V*. *mimicus* is also considered as the reservoir of the heat-stable toxin (ST) gene among the species of the genus *Vibrio* [39]. The *st* gene encoding ST of *V*. *mimicus* is identical to that of *V*. *cholerae* non-O1/non-O139. All isolates (*n* = 16) of *V*. *mimicus* in this study were isolated from the Min River in Fujian and were positive for *vmh* and *st* genes but negative for *tdh* and *hlx* genes. The *tdh* and *hlx* genes were demonstrated to only exist in clinical isolates [38,63], which could explain its absence in our aquatic isolates. The *vmh* gene, which can be identified in both clinical and environmental isolates, is thought to be a common gene of *V*. *mimicus* and a useful marker of identification of the species [38,63]. Compared to the *nag-st* gene in *V*. *cholerae*, the *st* gene detected in *V*. *mimicus* was more frequently [63]. (3) For *V*. *parahaemolyticus*, the major virulence factors are the thermolabile hemolysin encoded by *tl*, thermostable direct hemolysin encoded by *tdh*, and thermostable direct related hemolysin encoded by *trh* [40]. Our results suggested that all *V*. *parahaemolyticus* isolates (*n* = 14) contained *tl* gene. A study including 111 *V*. *parahaemolyticus* isolates from different origins found that all isolates possessed *tl* gene [40], indicating *tl* gene may be species-specific, as *vmh* gene for *V*. *mimicus*. The absence of *tdh* or *trh* gene in the aquatic isolates of *V*. *parahaemolyticus* in this study suggested they have no pathogenicity towards human because either *tdh* or *trh* gene is needed for clinical significance [64]. (4) For *V*. *harveyi*, many virulence factors such as caseinase, gelatinase, phospholipase, lipase, haemolysin, cysteine protease, metalloprotease, serine protease and chitinase, have been identified [41]. The *chiA* gene encoding chitinase, and *vhh* gene encoding hemolysin, were identified in all *V*. *harveyi* isolates (*n* = 13) in this study. The gene encoding serine protease was present in all isolates except one (*n* = 12). The high prevalence of *chiA*, *vhh*, and serine protease genes were consistent with previous studies [17,41]. *luxR* is the main regulatory gene of the signal transduction cascade of the *V*. *harveyi* quorum-sensing system [65] and was present in all *V*. *harveyi* isolates in our study, indicating the conserved quorum-sensing system of this species. ToxR, the gene product of *toxR*, is a transmembrane transcription regulator, which controls the coordinate expression of virulence genes in vibrios [66]. In this study, *toxR_Vh_* was absent in all *V*. *harveyi* isolates, which was different from previous studies, in which the high prevalence of *toxR_Vh_* gene was identified [17,41]. Interestingly, despite the similar virulence gene profiles, the *V*. *harveyi* isolates were collected from different regions and various aquatic environments. (5) For *V*. *fluvialis*, major virulence factors include hemolysin, protease, lipase and cytotoxin. The genes *vfh* encoding hemolysin, *hupO* encoding heme utilization protein and *vfpA* encoding protease, were identified in all isolates except one (*n* = 11). The high prevalence of these virulence gene was consistent with a previous study in which *vfh*, *hupO* and *vfpA* were detected in all *V*. *fluvialis* strains from patients and environment in China [9]. A recent study about the *Vibrio* isolates from rustic environmental freshwaters in South Africa found that the proportions of *hupO*, *vfpA* and *vfh* genes in *V*. *fluvialis* isolates were 14.6%, 0 and 19.5%, respectively [55]. Therefore, the virulence gene profiles in *V*. *fluvialis* isolates are likely to be influenced by geographic sites, weather pattern, habitat characteristics and other environmental factors.

Multilocus sequence typing (MLST) is an efficient tool to achieve the genetic characterization of bacterial pathogens [21] and has been applied to study the molecular evolution of members in *Vibrionaceae* [25]. In this study, the population structure and genetic diversity of 26 isolates was identified by MLST based on a scheme for *Vibrio* species [26]. Among the 26 isolates, only two isolates belonging to *V*. *cholerae* shared the same ST (ST_473_), and the remaining 24 isolates were divided into 24 different STs (ST_24_, ST_472_, ST_474–495_). The isolate Vi_36 of ST_24_ in PubMLST database, which belongs to *V*. *alginolyticus*/*diabolicus*, was sourced from live marine animals in Italy in 2007. The isolate H10 of ST_24_ in our study belongs to *V*. *diabolicus* based on the 16S rRNA sequence and was isolated from seawater in Qinhuangdao, Hebei Province in China. The differences indicates that this sequence type is not regional- or host-specific. The ST_472–495_ were identified for the first time, while the ST_24_ has been reported before. The new STs were due to the combination of new alleles or the new combination of previously reported alleles. The two *V*. *cholerae* isolates (B3 and B22) sharing the same ST (ST_473_) were collected from different freshwater rivers in Beijing, although the profiles of virulence genes were identical. However, the isolates (M1 and M6) of *V*. *mimicus* were collected from the same place and shared identical virulence gene profiles but showed very different allele combinations and thus STs. The isolates of other species, such as *V*. *parahaemolyticus*, despite the identical virulence gene profiles, were collected from different regions, and also showed different STs. Therefore, the diversity of STs identified in this study should be a complex interaction of many environmental factors and the rapid evolution of *Vibrio* isolates in aquatic habitats. *Vibrio* strains isolated from environment have been demonstrated to show a high degree of genetic diversity in previous studies [25,53].

Among the 143 isolates, only three isolates were shown to contain antibiotic-resistant genes analyzed in this study. The antibiotic-resistant genes were selected based on previous studies about *Vibrio* investigation [55,67]. Many different classes of antibiotics were included in order to identify multidrug resistant isolates. According to a study about the antibiotic resistance of *Vibrio parahaemolyticus* and *Vibrio vulnificus* in various countries, environmental and clinical *Vibrio* isolates were shown to share similar antibiotic resistance profiles [68]. Therefore, apart from antibiotics genes commonly seen in environmental *Vibrio* isolates, *tetA* gene encoding for tetracycline, which is commonly used to treat clinical *Vibrio* infection, was included. In this study, for most *V*. *cholerae* strains, the absence of antibiotic-resistant genes may because of the absence of the SXT element, which contributes to the trend of drug resistance [69]. Several aminoglycosides resistance genes *strA*, *strB*, *aadA*, *aph(3’)-Ia* (*aphA1*), two chloramphenicol resistance genes *cmlA1*, *floR* and one tetracycline resistance gene *tetA* were identified in strain B19, indicating that environmental isolates could be reservoirs of resistance genes [70]. The proportion of antibiotic-resistant gene containing isolates (2.1%, 3/143) in our study was lower than in previous studies focused on environmental *Vibrio* isolates. Resistance to cefazolin, ampicillin and imipenem was identified in 68.70%, 47.83% and 27.83% non-O1/non-O139 *V*. cholerae isolates from freshwater rivers in Zhejiang, China [53]. In addition, among all these isolates, 36.52% were defined as multidrug resistant [53]. Resistant genes encoding for aminoglycoside, phenicols, beta-lactams, carbapenems and fluoroquinolones were identified in most *Vibrio* isolates from rustic environmental freshwaters [55]. The prevalence of antibiotic-resistant genes is likely achieved through horizontal transfer from other bacteria strains in the aquatic environment [71]. Genetic interactions leading to the acquisition of plasmids, transposable elements, super-integron and integrating conjugative elements (ICEs) gene can confer antibiotic resistance to *Vibrio* species [72].

In conclusion, our study provides a comprehensive investigation of the *Vibrio* isolates from different aquatic environments in China in 2020, in terms of species composition, virulence gene distribution, sequence type, and antibiotic resistance. The findings suggest that aquatic *Vibrio* isolates are potential sources of human infection and show a high degree of genetic diversity, indicating that routine surveillance of aquatic *Vibrio* community will be of significance to public health.

## Figures and Tables

**Table 1:** Primers for virulence genes, MLST, and antibiotic-resistant genes.

Gene	Sequence (5′-3′)	Ta ^a^	Amplicon Size (bp)	References
*ctxAB*	F: CTCAGACGGGATTTGTTAGGCACG	55	302	[30]
	R: TCTATCTCTGTAGCCCCTATTACG			
*tcpA* (1) ^b^	F: GTGACTGAAAGTCATCTCTTC	55	1248	[31]
	R: AATCCGACACCTTGTTGGTA			
*tcpA* (2) ^b^	F: ATATGCAATTATTAAAACAGC	55	1052	[31]
	R: TTATTATTACCCGTTGTCGG			
*mshA*	F: CGCACAATGAGGTTCGCCAAG	60	512	[32]
	R: CCGAAAATTGACCGCCATTATC			
*hlyA* ^c^	F: GGCAAACAGCGAAACAAATACC	60	481	[33]
	R: CTCAGCGGGCTAATACGGTTTA			
*rtxC*	F: CGACGAAGATCATTGACGAC	55	263	[34]
	R: CATCGTCGTTATGTGGTTGC			
*rtxA*	F: CTGAATATGAGTGGGTGACTTACG	55	417	[34]
	R: GTGTATTGTTCGATATCCGCTACG			
IS1004	F: ATTGTCATCCCTAAACCACC	60	603	[35]
	R: AGGCGGTTTTAATATAAGCC			
*chxA*	F: TGTGTGATGATGCTTCTGG	52	2000	[36]
	R: TTATTTCAGTTCATCTTTTCGC			
SXT	F: TCGGGTATCGCCCAAGGGCA	60	946	[37]
	R: GCGAAGATCATGCATAGACC			
T3SS (*vcsV2*)	F: ATGCAGATCTTTTGGCTCACTTGATGGG	55	742	[35]
	R: ATGCGTCGACGCCACATCATTGCTTGCT			
*nag-st*	F: TATTATTTTCTTCAATCGCATTTAGC	60	206	[32]
	R: ATTTAAACATCCAAAGCAAGCTGG			
*vmh*	F: GGTAGCCATCAGTCTTATCACG	55	289	[38]
	R: ATCGTGTCCCAATACTTCACCG			
*tdh* (*V. mimicus*)	F: GGTACTAAATGGCTGACATC	55	251	[38]
	R: CCACTACCACTCTCATATGC			
*hlx*	F: CTGCCCATTAGAAACACCCT	55	382	[38]
	R: GTTGCTCATTCTCTGTCACC			
*st*	F: GAGAAACCTATTCATTGCA	50	216	[39]
	R: GCAAGCTGGATTGCAAC			
*tl*	F: AAAGCGGATTATGCAGAAGCACTG	58	450	[40]
	R: GCTACTTTCTAGCATTTTCTCTGC			
*tdh* (*V. parahaemolyticus*)	F: GTAAAGGTCTCTGACTTTTGGAC	58	269	[40]
	R: TGGAATAGAACCTTCATCTTCACC			
*trh*	F: TTGGCTTCGATATTTTCAGTATCT	58	500	[40]
	R: CATAACAAACATATGCCCATTTCCG			
*luxR*	F: ATGGACTCAATTGCAAAGAG	50	618	[41]
	R: TTAGTGATGTTCACGGTTGT			
*toxR_Vh_*	F: CGACAACCAAAATACGGAA	50	131	[41]
	R: AGAGCAATTTGCTGAAGCTA			
*chiA*	F: GGAAGATGGCGTGATTGACT	50	232	[41]
	R: GGCATCAATTTCCCAAGAGA			
serine protease	F: TGCACGACCAGTTGCTTTAG	50	232	[41]
	R: AAGTGGTCGTCAGCAAATCC			
*vhh*	F: TTCACGCTTGATGGCTACTG	50	234	[41]
	R: GTCACCCAATGCTACGACCT			
*vfh*	F: GCGCGTCAGTGGTGGTGAAG	61	800	[9]
	R: TCGGTCGAACCGCTCTCGCTT			
*hupO*	F: ATTACGCACAACGAGTCGAAC	56	600	[9]
	R: ATTGAGATGGTAAACAGCGCC			
*vfpA*	F: TACAACGTCAAGTTAAAGGC	55	1790	[9]
	R: GTAGGCGCTGTAGCCTTTCA			
DNA gyrase, β subunit (*gyrB*)	F: GAAGGTGGTATTCAAGCGTT	55	570	[26]
	R: CGGTCATGATGATGATGTTGT			
Uridylate kinase (*pyrH*)	F: CCCTAAACCAGCGTATCAACGTATTC	55	501	[26]
	R: CGGATWGGCATTTTGTGGTCACGWGC			
Recombinase A (*recA*)	F: TGCGCTAGGTCAAATTGAAA	55	462	[26]
	R: GTTTCWGGGTTACCRAACATYACACC			
ATP synthase, α subunit (*atpA*)	F: ATCGGTGACCGTCARACWGGTAAAAC	60	489	[26]
	R: ATACCTGGGTCAACCGCTGG			
*strA*	F: CTTGGTGATAACGGCAATTC	53	548	[42]
	R: CCAATCGCAGATAGAAGGC			
*strB*	F: ATCGTCAAGGGATTGAAACC	53	509	[42]
	R: GGATCGTAGAACATATTGGC			
*aadA*	F: GTGGATGGCGGCCTGAAGCC	68	525	[43]
	R: AATGCCCAGTCGGCAGCG			
*aac(3)-IIa* (*aacC2*)	F: CGGAAGGCAATAACGGAG	50	740	[44]
	R: TCGAACAGGTAGCACTGAG			
*aph(3’)-Ia* (*aphA1*)	F: ATGGGCTCGCGATAATGTC	50	600	[44]
	R: CTCACCGAGGCAGTTCCAT			
*aph(3’)-IIa* (*aphA2*)	F: GAACAAGATGGATTGCACGC	50	680	[44]
	R: GCTCTTCAGCAATATCACGG			
*cmlA1*	F: CACCAATCATGACCAAG	60	115	[45]
	R: GGCATCACTCGGCATGGACATG			
*catI*	F: AGTTGCTCAATGTACCTATAACC	50	547	[44]
	R: TTGTAATTCATTAAGCATTCTGCC			
*catII*	F: ACACTTTGCCCTTTATCGTC	50	543	[44]
	R: TGAAAGCCATCACATACTGC			
*floR*	F: CGCCGTCATTCCTCACCTTC	50	215	[44]
	R: GATCACGGGCCACGCTGTGTC			
*bla*_TEM_	F: TTTCGTGTCGCCCTTATTCC	60	690	[46]
	R: CCGGCTCCAGATTTATCAGC			
*sulI*	F: TTCGGCATTCTGAATCTCAC	50	822	[44]
	R: ATGATCTAACCCTCGGTCTC			
*dfrA1*	F: CGAAGAATGGAGTTATCGGG	60.5	372	[47]
	R: TGCTGGGGATTTCAGGAAAG			
*qnrA*	F: TTCAGCAAGAGGATTTCTCA	55	628	[48]
	R: GGCAGCACTATTACTCCCAA			
*tetA*	F: GCTACATCCTGCTTGCCTTC	55	210	[49]
	R: CATAGATCGCCGTGAAGAGG			

^a^ Annealing temperature; ^b^ Two pairs of *tcpA* primers were used. These two primer pairs have been used previously to amplify divergent *tcpA* alleles in the 7th pandemic *Vibrio* pathogenicity island (VPI) [31]; ^c^ The *hlyA* primers were used for *V. cholerae* O1 E1 Tor.

**Table 2:** The allelic profiles and STs of the analyzed isolates.

Strain No.	*gyrB*	*pyrH*	*recA*	*atpA*	ST
H10	22	20	22	16	24
B2	224	158	216	159	472
B3	223	159	217	160	473
B22	223	159	217	160	473
B12	225	160	218	161	474
M1	226	161	219	90	475
M6	227	162	220	90	476
M14	229	55	221	60	477
H3	228	36	52	19	478
W15	230	163	44	162	479
Y3	231	40	222	19	480
M21	232	164	223	163	481
H2	233	20	224	16	482
W6	112	17	225	129	483
Y1	74	108	107	16	484
H8	234	165	226	164	485
W7	235	166	227	165	486
Q3	236	167	228	166	487
W31	237	168	229	166	488
Q1	238	169	230	167	489
W5	240	170	231	169	490
B1	239	171	232	168	491
W1	241	172	233	170	492
H14	242	19	234	17	493
W20	243	19	138	17	494
Q12	244	173	235	171	495

## Data Availability

The datasets generated and analyzed during the current study are available from the corresponding author on reasonable request.
